# Fishing for novel HDAC inhibitor compounds to treat Duchenne muscular dystrophy

**DOI:** 10.1016/j.omtn.2025.102713

**Published:** 2025-09-22

**Authors:** Katherine G. English, Matthew S. Alexander

**Affiliations:** 1 Department of Pediatrics, Division of Neurology at the University of Alabama at Birmingham and Children’s of Alabama, Birmingham, AL 35294, USA; 2 UAB Center for Exercise Medicine at the University of Alabama at Birmingham, Birmingham, AL 35294, USA; 3 UAB Civitan International Research Center (CIRC), at the University of Alabama at Birmingham, Birmingham, AL 35233, USA; 4 UAB Center for Neurodegeneration and Experimental Therapeutics (CNET), Birmingham, AL 35294, USA

**Figure:**
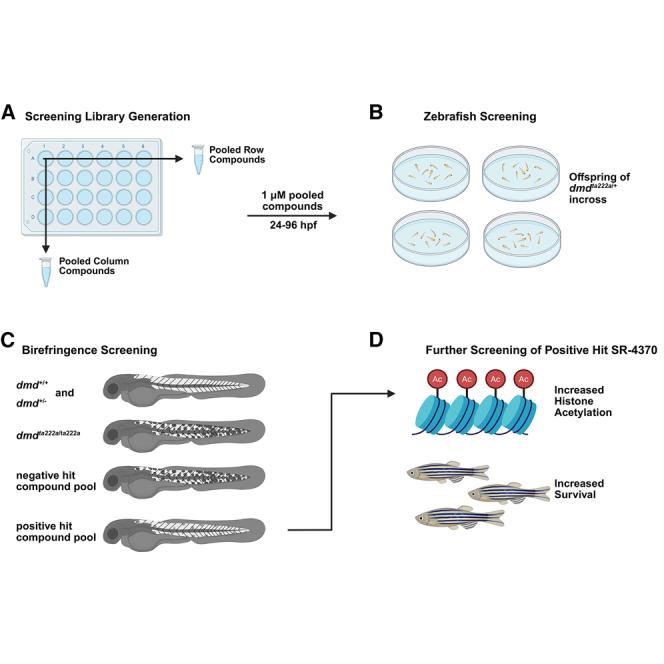

Duchenne muscular dystrophy (DMD) is a debilitating neuromuscular disorder caused by pathogenic variants resulting in the absence of a functional dystrophin protein. Patients with DMD have progressive muscle weakness, and are usually non-ambulatory by their teenage years. As DMD progresses, skeletal respiratory muscles fail and patients with DMD will often develop dilated cardiomyopathy leading toward early death on average in their third decade of life. Glucocorticoids can maintain skeletal limbal and respiratory muscle strength, and promising dystrophin-replacement strategies have shown efficacy in mitigating symptoms. More recently, an histone deacetylase (HDAC) inhibitor compound (givinostat; DUVYZAT; manufactured by Italfarmaco) has gained US Food and Drug Administration (FDA) approval in patients with DMD for its ability to reduce inflammation while preventing muscle loss. Nevertheless, all of these strategies address aspects of DMD pathophysiologies, but do not cure the ultimate outcome of the disease.

The authors of the Louie et al. study sought to identify novel corrective HDAC inhibitors (HDACi) compounds that could correct DMD muscle symptoms using a dynamic drug library screening approach and model, the zebrafish.^1^ Zebrafish are a powerful animal model that serve as valuable models for phenotypic analysis and, more importantly, drug screening in zebrafish.^2^ The *sapje* (*dmd*^*ta222a*^) mutant harbors an exon 4 mutation in the zebrafish (*dmd*) gene and display severe dystrophic pathologies.^3^ The *sapje* mutant zebrafish have served as a valuable platform for identifying corrective epigenetic drug compounds using commercial and academic drug compound libraries.^4^ The *sapje* mutant larval zebrafish display poor skeletal muscle birefringence, impaired locomotion, and progressive muscle damage leading toward early lethality. These phenotypic traits can be individually or combinatorially screened as biomarkers to determine if a drug is effective at correcting a specific aspect of DMD disease pathology. More recent in-depth analysis of the *sapje* zebrafish has identified physiological force deficits, impaired mitochondria dynamics, altered kinematic movement, and a dynamic platform for studying *in vivo* protein dynamics.^5^^,^^6^ These factors along with the ability of larvae zebrafish to uptake corrective drug compounds in their skin and gills, demonstrated the increased usage of the *sapje* zebrafish as an important platform for high-throughput drug library evaluation.

The Louie et al. authors sought to advance on their previous work demonstrating that the class I/II HDAC inhibitor trichostatin A (TSA) ameliorated dystrophic muscle birefringence in *dmd* morphant (*dmd* MO) zebrafish.^7^ A previous epigenetic drug library screen of the authors identified two HDACi (oxamflatin and salermide) that ameliorated dystrophic myofiber breakdown in the *sapje* mutants.^4^ The authors in the present study used a commercially available epigenetic drug compound library (MedChemExpress) of 817 compounds sorted into 185 unique drug pools, and evaluated the ability of each drug compound to correct skeletal muscle birefringence from 24 to 96 h post-fertilization (hpf) in embryos from *dmd*^*ta222a*/+^ heterozygote matings (Figure 1). The authors compared their epigenetic drug hits from the screen with the positive controls TSA and givinostat, an FDA-approved drug for DMD boys ages 6 and older. In DMD clinical trials, givinostat improved muscle flexion and prevented muscle functional declines in ambulatory DMD boys.^8^ The current authors identified one epigenetic molecule in particular, SR-4370, that both reduced abnormal muscle birefringence and extended lifespan in the *sapje* mutant zebrafish in secondary drug evaluation. SR-4370 had previously been identified in a high-throughput screen for drug compounds that reactivated latent HIV-1 viral reservoirs toward the development of antiretroviral therapies.^9^ Furthermore, the authors also determined that HDACi treatments that improved overall muscle histopathologies in *dmd* zebrafish caused increased histone acetylation in HDACi-treated larvae. The authors concluded that HDACi are a promising avenue for the overall treatment of DMD-associated skeletal muscle symptoms.

**Figure 1:**
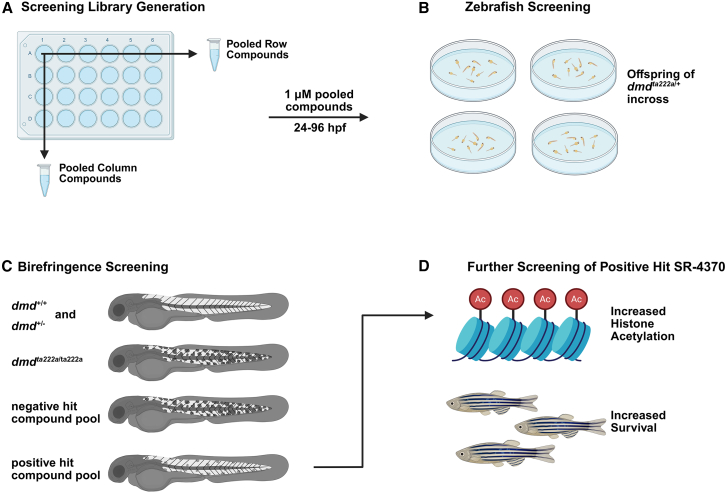
Screening of an epigenetic drug compound library in *dmd* mutant zebrafish (A) Graphical abstract highlighting the key steps in the *sapje* zebrafish larvae drug screen. The screening platform consisted of the 817 drug compound library using 180 pooled compounds. (B) A screening dose of 1 μM was used to evaluate the offspring from the *dmd*^*ta222a*/+^ heterozygote matings 24–96 h post-fertilization (hpf). (C) Skeletal muscle birefringence imaging evaluated in the offspring from the *dmd*^*ta222a*/+^ heterozygote matings. (D) Additional follow up screening of the hit compound SR-4370 showed increased histone acetylation and lifespan in the *dmd*^*ta222a*/*ta222a*^ homozygote mutant zebrafish.

There are several strengths and weaknesses from the Louie et al. study that should be considered to fully evaluate the utilization of this approach and HDACi’s for the treatment of DMD. One significant weakness is the authors focused solely on the skeletal muscle pathologies associated with DMD, and other affected DMD tissues (namely, the heart, brain, and smooth muscle) were not evaluated for improved pathologies. HDAC inhibitors have shown promise in improving skeletal muscle strength via blocking mitochondria defects, reducing muscle fibrotic content, and increasing muscle mass in animal models; however, the exact mechanism of action of many of these HDACi’s in DMD disease is not fully elucidated.^10^ The drug screen itself was also performed during the early embryonic stage of the developing zebrafish larvae, something that would be very difficult if not impossible to evaluate *in utero* in a DMD human embryo. Zebrafish as a model for DMD has some limitations including a differential immune cellular repertoire, three-chambered hearts, and different muscle oxygen requirements compared to mammalian muscles. Furthermore, expanded follow up studies of the specific lead HDACi SR-4370 including drug toxicology, pharmacokinetics (PK)/pharmacodynamics (PD) evaluation, and long-term testing in mammalian and eventual human patient studies are required for advancing this particular HDACi into DMD patient use. Nevertheless, this study is an excellent addition to the growing library of studies using zebrafish drug screening for rare human diseases. Additionally, it confirms the notion that HDAC inhibition is effective in blocking the onset of dystrophic skeletal muscle symptoms and is beneficial at preventing DMD-associated myofiber loss. One can envision a prescribed treatment of an HDAC inhibitor to strengthen dystrophin-deficient muscles in combination with a DMD gene therapy viral vector that blocks myofiber breakage to improve outcomes for DMD boys.

## Acknowledgments

M.S.A. is supported by NIH grants from the Office of the Director under award number (U54OD030167), an R21 grant from NINDS (R21NS140497), and the Department of Defense Congressionally Directed Medical Research Program (DoD Congressionally Directed Medical Research Programs [CDMRP]) grant award (MD240021). K.G.E. is supported by an NIH NICHD training grant (T32HD071866).

## Declaration of interests

The authors declare no competing interests.
